# Atrial function and geometry differences in transthyretin versus immunoglobulin light chain amyloidosis: a cardiac magnetic resonance study

**DOI:** 10.1038/s41598-021-03359-9

**Published:** 2022-01-07

**Authors:** Cassady Palmer, Vien T. Truong, Jeremy A. Slivnick, Sarah Wolking, Paige Coleman, Wojciech Mazur, Karolina M. Zareba

**Affiliations:** 1 The Christ Hospital Health Network, 2123 Auburn Ave, Ste 138, Cincinnati, OH 45219 USA; 2 The Lindner Research Center, Cincinnati, OH USA; 3 The Ohio State University Wexner Medical Center, Columbus, OH USA

**Keywords:** Cardiology, Cardiovascular diseases

## Abstract

To determine the differences in left atrial (LA) function and geometry assessed by cardiac magnetic resonance (CMR) between transthyretin (ATTR) and immunoglobulin light chain (AL) cardiac amyloidosis (CA). We performed a retrospective analysis of 54 consecutive patients (68.5% male, mean age 67 ± 11 years) with confirmed CA (24 ATTR, 30 AL) who underwent comprehensive CMR examinations. LA structural and functional assessment including LA volume, LA sphericity index, and LA strain parameters were compared between both subtypes. In addition, 15 age-matched controls were compared to all groups. Patients with ATTR-CA were older (73 ± 9 vs. 62 ± 10 years, p < 0.001) and more likely to be male (83.3% vs. 56.7%, p = 0.036) when compared to AL-CA. No significant difference existed in LA maximum volume and LA sphericity index between ATTR-CA and AL-CA. LA minimum volumes were larger in ATTR-CA when compared with AL-CA. There was a significant difference in LA function with worse strain values in ATTR vs AL: left atrial reservoir [7.4 (6.3–12.8) in ATTR vs. 13.8 (6.90–24.8) in AL, p = 0.017] and booster strains [3.6 (2.6–5.5) in ATTR vs. 5.2 (3.6–12.1) in AL, p = 0.039]. After adjusting for age, LA reservoir remained significantly lower in ATTR-CA compared to AL-CA (p = 0.03), but not LA booster (p = 0.16). We demonstrate novel differences in LA function between ATTR-CA and AL-CA despite similar LA geometry. Our findings of more impaired LA function in ATTR may offer insight into higher AF burden in these patients.

## Introduction

Cardiac amyloidosis (CA) is one of the most prevalent infiltrative cardiomyopathies and is chiefly responsible for the ultimate prognosis in patients with systemic amyloid deposition^1^. CA most frequently occurs when misfolded aggregates of either immunoglobulin light chains (AL) amyloidosis or transthyretin (ATTR) amyloidosis deposit within cardiac tissue^2,3^. The misfolded aggregates of both light chain and ATTR are believed to initiate a cytotoxic cascade within cardiac myocytes^4–7^. Amyloid fibril deposition initiates myocardial interstitial expansion leading to contractile dysfunction finally culminating in organ failure^8^. This interstitial expansion and contractile dysfunction are not limited to ventricular chambers but can also demonstrate atrial manifestations. Atrial enlargement has been commonly reported within CA populations. Atrial volume and tissue characterization by late gadolinium enhancement (LGE) cardiovascular magnetic resonance (CMR) assessment have been frequently used to quantify amyloid fibril burden. However, LGE interpretation can be susceptible to misinterpretation as intrinsic factors such as presence of patchy focal LGE and suboptimal myocardial nulling may vastly underestimate the degree of involvement^9^.

It is important to highlight that while atrial size is most frequently reported, it is not in itself equivalent to atrial function^10^. Atrial function is dynamic with tripartite physiology comprised of reservoir, conduit, and booster phases. During the reservoir phase, the left atrium serves as a distensible chamber accepting blood from pulmonary veins, the conduit phase correlates to the passive filling of the left ventricle, and the booster phase completes the cycle with atrial contraction^11^. CMR yields high spatial resolution which is beneficial in assessment of thin atrial wall tissue. Recent CMR techniques incorporating feature tracking allow for accurate and reproducible capture of dynamic left atrial function^12^. Furthermore, LA enlargement fails to capture maladaptation throughout continuum of the disease process since enlargement is primarily identified as a late manifestation of pathology. Early detection of CA is therefore instrumental to initiate appropriate therapy^1^. We sought to investigate atrial function and structure in patients with CA utilizing CMR.

## Results

### Baseline patient characteristics

In total, 54 patients with cardiac amyloidosis who had adequate CMR image quality for LA strain analysis were included in the study (68.5% male, mean age 67 ± 11 years). There were 30 patients with AL and 24 patients with ATTR cardiac amyloidosis. Clinical characteristics are presented in Table 1. In addition, 15 age-matched healthy controls are compared to further elucidate differences (53% male, mean age of 61 ± 6 years). Compared to AL-CA, patients with ATTR-CA were older (73 ± 9 vs. 62 ± 10, p < 0.001) and more likely to be male (83.3% vs. 56.7%, p = 0.036). There were no significant differences in the rates of hypertension, hyperlipidemia, diabetes, and NYHA Class between the groups (p > 0.05 for all, Table 1).

**Table 1:** Baseline clinical characteristics.

	Overall (n = 54)	AL-CA (n = 30)	ATTR-CA (n = 24)	p value
Age, years	67 ± 11	62 ± 10	73 ± 9	< 0.001
Male (%)	37 (68.5)	17 (56.7)	20 (83.3)	0.036
**NYHA (%)**				0.16
I	3 (5.6)	3 (10.0)	0 (0)	
II	23 (42.6)	13 (43.3)	10 (41.7)	
III	19 (35.2)	9 (30)	10 (41.7)	
IV	3 (5.6)	3 (10)	0 (0)	
Creatinine (mg/dL)	1.18 ± 0.43	1.12 ± 0.47	1.26 ± 0.38	0.23
GFR (mL/min/1.73 m^2^)	71.7 ± 27.7	75.2 ± 31.5	67.2 ± 18.9	0.28
Hematocrit (%)	38.4 ± 4.9	37.8 ± 4.4	39.1 ± 5.5	0.33
BNP (ng/L)	371 (239–715)	432 (236–977)	345 (236–653)	0.65
Troponin (ng/mL)	0.16 (0.05–0.31)	0.11 (0.04–0.24)	0.19 (0.08–0.35)	0.19
Hypertension (%)	28 (52.8)	12 (41.4)	16 (66.7)	0.07
Hyperlipidemia	28 (52.8)	14 (48.3)	14 (58.3)	0.47
Diabetes (%)	9 (17)	3 (10.3)	6 (25)	0.27

Continuous variables are expressed as mean ± standard deviation or median (interquartile range). Categorical variables are presented as n (%).

*GFR* glomerular filtration rate, *NYHA* New York Heart Association.

### CMR parameters

Patients with ATTR-CA had larger left and right ventricular volumes and lower left and right ventricular ejection fraction as compared to AL-CA patients (Table 2). Extracellular volume trended towards significance with higher values in ATTR-CA vs AL-CA (53 ± 10 vs. 47 ± 10, p = 0.06).

**Table 2:** CMR Characteristics.

	Overall ( = 54)	AL-CA (n = 30)	ATTR-CA (n = 24)	p value*	Normal control (n = 15)	p value^ǂ^
LVEDVI (mL/m^2^)	73.4 ± 18.5	68.3 ± 17.4	80.1 ± 18.1	0.03	70.7 ± 14.8	0.64
LVESVI (mL/m^2^)	38.6 ± 15.8	32.3 ± 12.1	46.7 ± 16.7	0.002	29.8 ± 8.1	0.04
SVI (mL/m^2^)	34.0 (26.9–40.5)	36.3 ± 12.3	35.8 (26.5–39.5)	0.73	41.0 ± 10.5	0.045
LVEF (%)	49 ± 12	53 ± 11	43 ± 11	0.003	58 ± 8	0.006
LVMI (g/m^2^)	97.5 ± 27.7	93.0 ± 26.4	103.3 ± 28.9	0.21	48.8 ± 13.4	< 0.001
RVEDVI (mL/m^2^)	72.7 ± 18.9	68.2 ± 16.6	80.7 ± 20.5	0.03	65.0 ± 12.7	0.14
RVESVI (mL/m^2^)	39.5 ± 16.1	33.2 ± 11.2	46.9 ± 16.1	0.002	28.3 ± 8.5	0.001
RVSVI (mL/m^2^)	33.2 ± 10.4	33.6 ± 10.4	36.4 ± 13.2	0.41	36.6 ± 8.4	0.25
RVEF (%)	47 ± 13	51 ± 12	41 ± 12	0.007	57 ± 9	0.006
ECV (%)	50 ± 11	47 ± 10	53 ± 10	0.06	–	–
LA volume_max_ (mL)	83.6 ± 25.5	81.2 ± 24.6	86.6 ± 26.8	0.42	79.9 ± 10.1	0.61
LA volume index_max_ (mL/m^2^)	42.6 ± 12.1	42.2 ± 11.3	43.1 ± 13.4	0.78	41.2 ± 8.3	0.68
LA volume_min_ (mL)	63.5 ± 26.5	56.9 ± 24.5	71.8 ± 27.1	0.04	33.8 ± 9.7	< 0.001
LA volume index_min_ (mL/m^2^)	32.1 ± 12.3	29.4 ± 11.4	35.4 ± 12.8	0.07	17.4 ± 4.2	< 0.001
LAEF (%)	22.9 (14.2–37.2)	31.7 ± 17.8	18.6 ± 10.9	0.002	57.8 (56.1–60.5)	< 0.001
LA sphericity index	0.63 ± 0.15	0.64 ± 0.13	0.63 ± 0.18	0.91	0.64 ± 0.15	0.90
RA volume_max_ (mL)	72.1 (50.7–102.1)	74.9 ± 29.2	81.9 ± 45.1	0.50	48.4 ± 20.8	0.002
RA volume index_max_ (mL/m^2^)	36.5 (26.5–50.0)	38.8 ± 13.6	39.9 ± 20.0	0.82	22.2 (19.1–28.3)	0.001
RA volume_min_ (mL)	51.3 (29.6–80.3)	54.3 ± 28.5	55.2 (33.0–84.9)	0.65	25.3 ± 10.2	< 0.001
RA volume index_max_ (mL/m^2^)	26.5 (15.8–40.9)	27.9 ± 13.5	31.0 ± 18.9	0.50	12.0 (8.9–15.0)	< 0.001
LA booster (%)	4.6 (3.4–9.2)	5.2 (3.6–12.1)	3.6 (2.6–5.5)	0.039	17.9 (15.5–18.9)	< 0.001
LA conduit (%)	6.6 (4.0 – 11.9)	8.9 (4.1–14.4)	5.1 (3.7–8.3)	0.18	17.8 (15.6–22.9)	< 0.001
LA reservoir (%)	11.4 (6.8–17.9)	13.8 (6.90–24.8)	7.4 (6.3–12.8)	0.017	34.6 (32.8–44.0)	< 0.001

Continuous variables are expressed as mean ± standard deviation or median (interquartile range). Categorical variables are presented as n (%).

*LV* left ventricular, *RV* right ventricular, *LVEDVI* left ventricular end-diastolic volume index, *LVESVI* left ventricular end-systolic volume index, *SVI* stroke volume index, *EF* ejection fraction, *RVEDVI* right ventricular end-diastolic volume index, *RVSVI* right ventricular end-systolic volume index, *LA* left atrial, *ECV* extracellular volume, *EF* ejection fraction, *RA* right atrial.

*AL-CA vs. ATTR-CA.

^ǂ^CA vs. Normal control.

**Figure 1:**
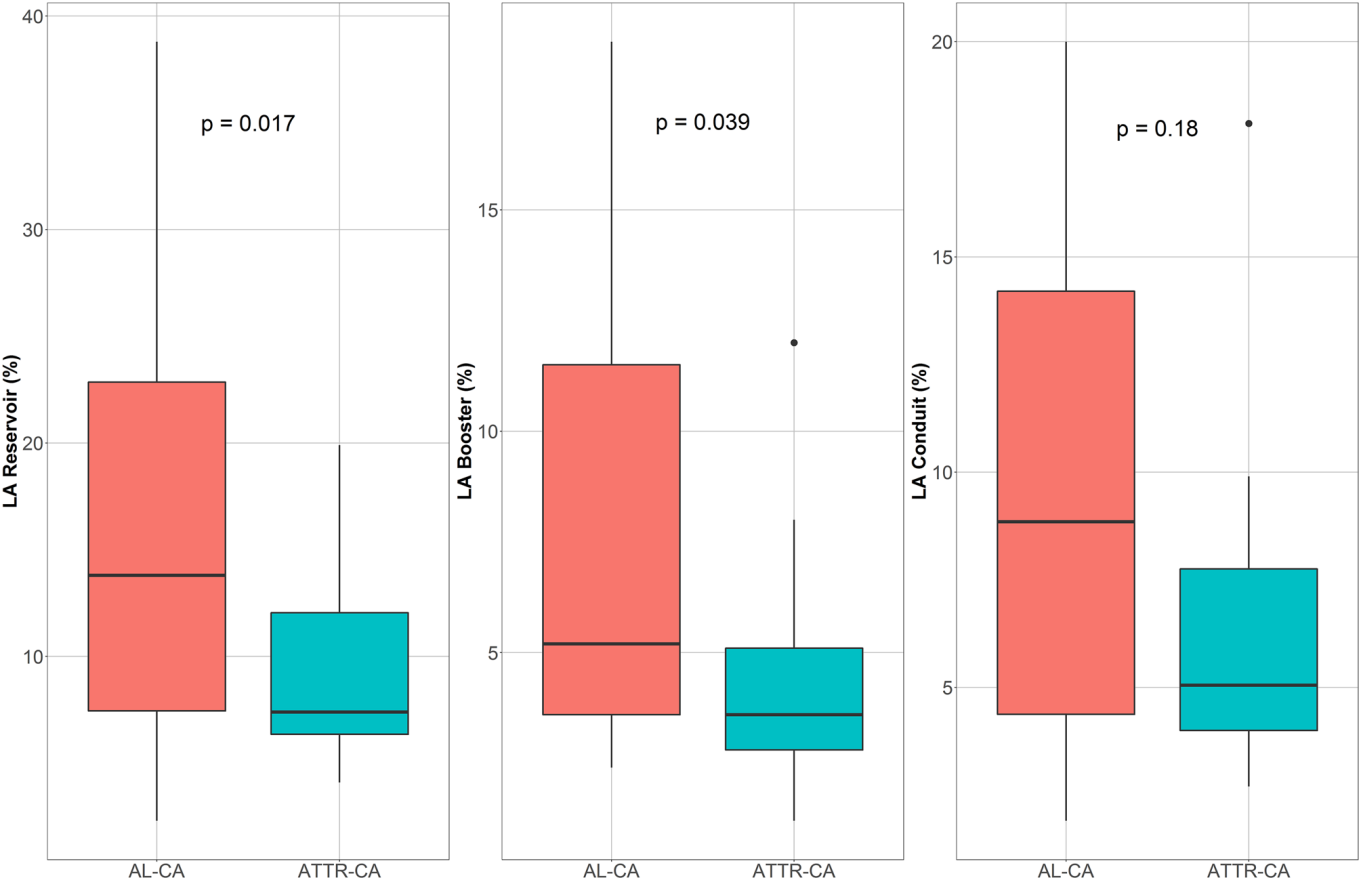
Box-and-whisker plot showing ATTR had significantly lower LA strain compared to AL.

Regarding atrial geometry, our study found maximal left atrial and right atrial volumes, and LA sphericity were comparable between two groups. However, minimal left atrial volume was significantly larger in ATTR-CA when compared with AL-CA (Table 2) leading to lower LA ejection fraction in ATTR-CA. Patients with ATTR-CA has significantly worse LA reservoir [7.4 (6.3–12.8) in ATTR vs. 13.8 (6.90–24.8) in AL, p = 0.017] (Fig. 1) and LA booster strains [3.6 (2.6–5.5) in ATTR vs. 5.2 (3.6–12.1) in AL, p = 0.039] as compared to AL-CA patients. Conduit strain analysis did not reach significance upon group comparison. After adjusting for age, LA reservoir remained significantly lower in ATTR-CA compared to AL-CA (p = 0.03), but not LA booster (p = 0.16). When comparing our CA cohorts to 15 healthy age and gender matched volunteers, we found that LA volumes were markedly larger in CA and LA function was significantly worse in CA (Table 2). Furthermore, there are 10 ATTR-CA patients with wild-type and 14 remaining with mutation. Age difference was significantly observed between the two groups (78.0 ± 9.5 for wild-type ATTR-CA vs. 70.4 ± 6.8 for mutation, p = 0.04). After adjusting for age, wild-type ATTR-CA is not significantly different when compared to mutated ATTR-CA in terms of LA reservoir strain [6.4 (6.0–10.6) vs. 9.7 (7.2–14.1), p = 0.71], LA conduit strain [3.9 (3.0–8.5) vs. 5.1 (4.6–8.7), p = 0.74], and LA booster strain [3.6 (2.9–3.7) vs. 4.6 (2.4–7.5), p = 0.75].

## Discussion

We demonstrate novel findings in LA function and geometry between CA subtypes. We note that (1) CMR feature tracking revealed significant functional differences between CA subtypes with lower LA function in patients with ATTR vs AL, despite (2) no difference in LA geometry. Finally our study provides observational insight into a potential explanation of underlying mechanisms responsible for previously reported differences in prevalence of atrial fibrillation (AF) based upon CA subtype which have yet to be fully elucidated^13^.

The LA contributes to the modulation of LV filling, and maladaptive changes to the LA have been shown to prognosticate poor outcomes in patients with heart failure with preserved ejection fraction (HFpEF)^14^. The comprehensive assessment of LA function in CA patients is underscored by the prognostic implication of worsening impairment, as HFpEF is a common clinical characteristic. LA function is tripart and reported as (1) reservoir, (2) conduit, and (3) booster strain. LA reservoir strain is commonly reported as it is most representative of chamber compliance. However, it should not go without mention that the remaining components (conduit and booster strain) are the summation of the reservoir, and the role of each in compensation when one fails should not be overlooked. We demonstrate differences in both LA reservoir and booster strain and a trend in LA conduit strain. Booster function has been previously reported to increase significantly as a compensatory mechanism in the aging population to maintain reservoir function^10^. In our CA cohort we noted that LA booster function was significantly impaired and did not compensate for decreased reservoir function. Although, the loss of LA compliance in CA has been explained as obligatory in response to direct amyloid fibril deposits in atrial walls limiting atrial stretch^15,16^ an apparent reduced booster strain may also be an integral parameter to consider. Of interest, a recent study with a large cohort of ATTR cardiomyopathy found atrial electromechanical dissociation in the presence of sinus rhythm within a small proportion of patients. The prognosis for patients with atrial electromechanical dissociation was significantly worse than for patients found with effective booster function and was comparable with patients in atrial fibrillation^16^. In view of new medical therapies recently introduced to block specific stages of amyloidogenesis, there is an opportunity to further investigate LA phasic changes and how these coincide with medical management. This was highlighted by Marwick and colleagues in a recent review when commenting on the additive value of LA functional assessment when monitoring of effects of medical management^14^.

Our study did not reveal any significance differences in LA geometry between CA subtypes when classified by sphericity indices and volumetric assessment. The morphological nature of the LA under normal loading conditions is that of a discoid shape; however, Bisbal et al. hypothesized that in response to increased LA wall stress/tension spherical remodeling occurs in order to alleviate wall stress/tension while accommodating for increased volumes^17^. These authors investigated LA geometrical remodeling by sphericity indices in patients undergoing AF ablation; however, there have been conflicting results when utilizing LA sphericity indices with many studies reporting this parameter to be isolated to the AF population^18^. Our findings reveal no significant difference in LA sphericity between CA subtypes. In addition, LA maximum volume index did not differ between both subtypes thus serving as another metric demonstrating absence of geometrical differences.

Finally, LA strain assessment has previously been utilized in risk stratification in a broad spectrum of cardiovascular diseases^19–22^. AF is a common risk factor in ATTR-CA with a reported occurrence in 70% of patients^23^. In an echocardiographic investigation of confirmed ATTR-CA patients Henein et al. discovered that the most sensitive predicator of atrial arrhythmia was LA strain rate during atrial contraction (LASRa)^24^. It has been reported that there is no correlation between LASRa and LA size, an observation indicating that morphological adaptation (i.e. LA enlargement) is not required for AF in ATTR patients^24^. While it appears that AF in this population does not increase mortality, clinical management remains challenging^25,26^. Shih et al. noted that AF patients with a history of stroke had significantly impaired LA reservoir strain rates compared to patients without stroke, concurrently demonstrating no differences in LA volume index^27^. Furthermore, in absence of AF it has been recently reported that isolated LA dysfunction has significant association with cardioembolic stroke^28^. These findings may carry significant implications for CA patients in the absence of AF and may offer explanation for higher reported rates of thromboembolic events. Once more this underscores the integral role of LA strain assessment in the evaluation of CA patients. Although our current study is only able to provide a concept for explaining the underlying mechanisms responsible for higher prevalence of AF in ATTR-CA, prior studies provide additional context for our data and an additional rationale for further investigations of atrial function. Moreover, our study revealed no significant geometrical differences between both ATTR-CA and AL-CA subtype; however, significant impairment of function as assessed by strain proved to be integral in unmasking pathophysiology which may have otherwise been overlooked. In addition, wild-type ATTR-CA is not significantly different when compared to mutation in terms of LA strain including LA reservoir, LA conduit, and LA booster.

This is a retrospective study thus it may be possible that other covariates offering further explanation in CA subtype differentiation may have been excluded. A major limitation to our current study was our inability to completely assess disease burden as well as incidence of AF over time between ATTR-CA and AL-CA subtypes given the small sample size. Future studies incorporating larger cohorts will help to further elucidate these differences. However, previous studies have demonstrated higher incidence of AF in ATTR-CA compared to AL-CA^23,25^. Of mention, prior literature on left atrial functional assessment in CA has primarily been conducted using echocardiographic assessment, we do not have echocardiographic comparisons for this current study. However, CMR is considered the gold standard in cardiac chamber quantification and we believe this serves as an advantage in the case of robust assessment of LA volumes and sphericity indices. Moreover, this current study offers a concept in the explanation of a potential underlying mechanism responsible for higher prevalence of AF in ATTR-CA utilizing CMR to assess functional as well as geometrical differences between the two CA subtypes.

## Methods

### Study participants

We performed a retrospective analysis of consecutive patients with confirmed CA who underwent comprehensive CMR exams between 2010 and 2019. Inclusion criteria included patients in sinus rhythm at the time of CMR, with adequate image quality for structural and strain analysis with confirmed CA. Diagnostic criteria for cardiac amyloidosis were defined as a positive endomyocardial biopsy, or a positive extracardiac biopsy with characteristic CMR features of cardiac involvement, or grade ≥ 2 myocardial involvement on Tc-PyP scan^29^. Biopsies were considered positive based on positive Congo red staining or immunohistochemistry. Expert Consensus Recommendations for Multimodality Imaging in Cardiac Amyloidosis were utilized to define characteristic CMR features^30^. AL-CA subtype was diagnosed by the presence of AL fibrils—either by immunohistochemistry or mass spectroscopy—on endomycardial biopsy or on biopsy of a non-cardiac site with characteristic cardiac imaging features^30^. ATTR-CA subtype was determined by the presence of TTR fibrils on endomyocardial biopsy or an extracardiac site with characteristic cardiac imaging features^30^. Additionally, ATTR-CA could be diagnosed non-invasively if there was grade 2–3 uptake on Tc-PyP in the absence of a monoclonal light chain protein on comprehensive serum and urine analysis. Within the ATTR-CA subtype 10 patients were confirmed wild type and 14 were confirmed mutation. The spectrum of mutation consisted of 11 patients with Val122Ile mutation, 2 patients with Thr60Ala mutation, and 1 patient mutation type was not recorded.

### Clinical data

Clinical characteristics and comorbidities were established by review of the medical record. The following baseline clinical characteristics were collected: age, gender, ethnicity, height, weight, glomerular filtration rate (GFR), hematocrit, troponin, and b-type natriuretic peptide (BNP). Information on comorbidities was queried from the medical chart including the presence of hypertension, diabetes, hyperlipidemia, and New York Heart Association Class (NYHA). The Ohio State University Institutional Review Board approved this retrospective study and waived informed consent. All methods and protocols for this study were performed in accordance with relevant guidelines and regulations.

### CMR protocol

**Figure 2:**
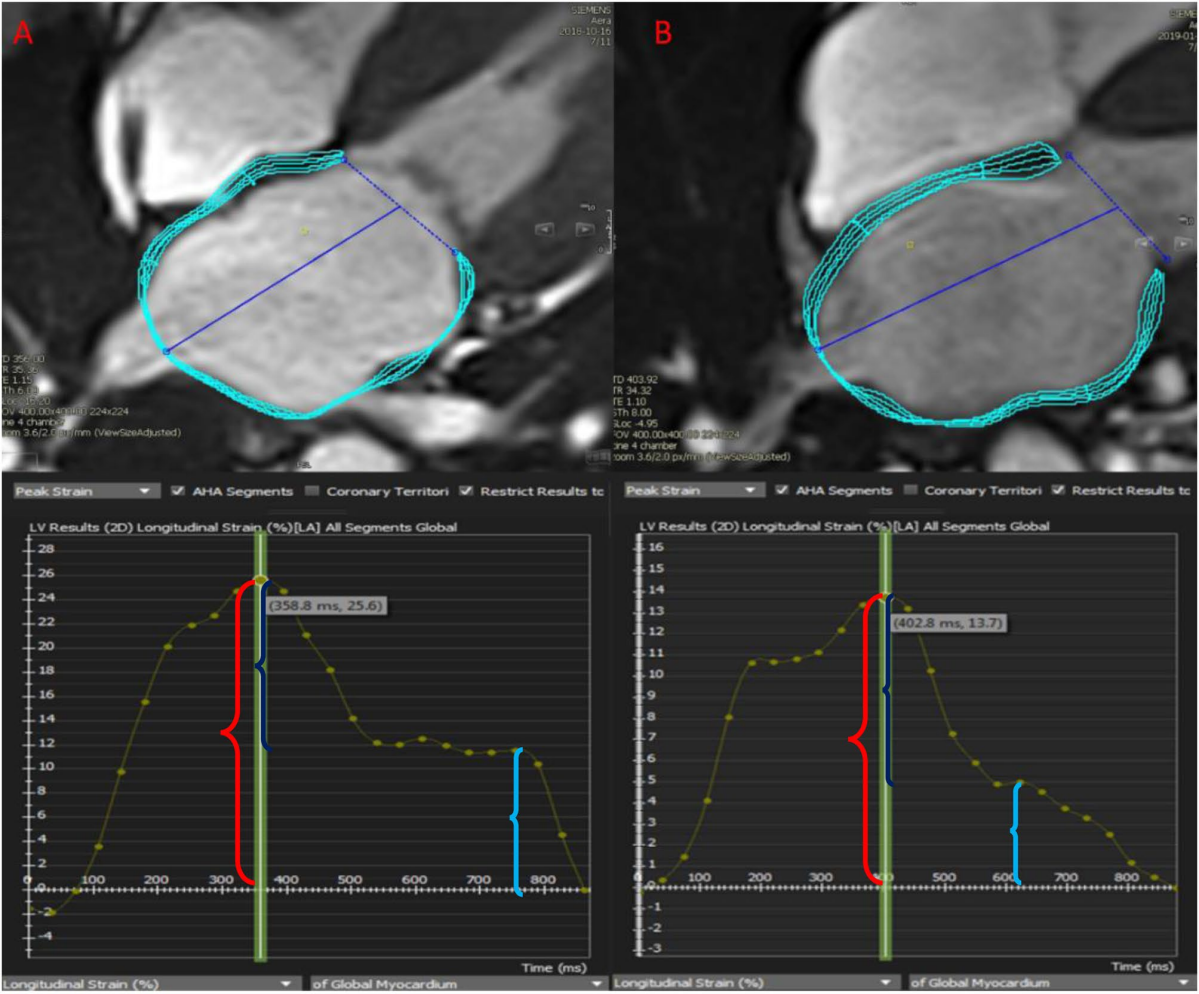
Global longitudinal left atrial (LA) strain curves (**A**) 60-year-old male diagnosed with AL amyloid with LV ejection fraction of 63%, LA reservoir strain = 26%, LA booster strain = 12%. (**B**) 65-year-old male diagnosed with ATTR amyloid with LV ejection fraction of 56%, LA reservoir strain = 14% LA booster strain = 4%. Red bracket parenthesis: Reservoir strain; light green bracket parenthesis: booster strain; blue bracket parenthesis: conduit strain.

All patients underwent clinical CMR scans with a 1.5 Tesla scanner (Magnetom Avanto or Espree, Siemens Medical Solutions, Erlangen, Germany). Steady-state free precession sequences (SSFP) were used for assessment of LV and RV volumes, EF and LV mass. Ventricular volumes and EF were measured from contiguous short-axis cine images using semi-automated software for endocardial segmentation using endocardial and epicardial contours at end-systole and end-diastole with Simpson’s rule. LV mass was calculated from the total end-diastolic myocardial volume multiplied by the specific gravity of the myocardium (1.05 g/mL)^31^. Atrial volumetric assessment was made from both horizontal and long axis cine SSFP sequences. LA maximum volume was traced using semi-automated software (Biplane, CMR42, Circle Cardiovascular Imaging Inc. Calgary, Alberta, Canada) in atrial diastole, and LA minimum volume was traced respectively in a similar fashion in atrial systole. Likewise, LA feature tracking was performed with utilizing both horizontal and vertical long axis cine SSFP acquisitions on dedicated software (Tissue tracking, CMR42, Circle Cardiovascular Imaging Inc. Calgary, Alberta, Canada). LA endocardial and epicardial contours were manually traced, and care was taken to exclude pulmonary veins and left atrial appendage insertions as illustrated in Fig. 2. Strain analysis was initiated during the diastolic phase and manually adjusted when tracking was suboptimal. LA sphericity index was calculated as the ratio of LA maximum volume to LA volume of a sphere with maximum LA length diameter from the two- and four-chamber images^32^.

LGE imaging was performed using a gradient-echo inversion recovery sequence with magnitude and phase sensitive inversion recovery reconstructions 10 min after standard dose of gadolinium-based contrast agent^33^. The presence of ventricular LGE was assessed by 2 expert level 3 trained operators blinded to clinical data and had to be present in either two consecutive short axis slices or in two orthogonal imaging planes. Modified Look-Locker Inversion Recovery (MOLLI) acquisition schemes were used to acquire T1 maps produced using vendor software before and 15 min after administration of contrast. T1 values and ECV were measured and calculated utilizing interventricular septal values from the mid short axis view. The region of interest was placed in the mid myocardium with manual tracing to avoid partial volume effects^9,34^. Myocardial ECV was calculated as previously described^35^. In patients with advanced renal dysfunction (GFR < 30 mL/min/1.73 m^2^) in whom gadolinium was not administered, only pre-contrast native T1 was assessed. In age matched controls, gadolinium was not administered.

### Statistical analysis

Categorical data are presented as frequency with percentage, and comparison between groups was done using the chi-square test or Fisher exact test as appropriate. The distribution of continuous variables was assessed using the histogram and QQ plot. Continuous variables are presented as mean ± standard deviation (SD) for normal distribution or as median (interquartile range) for non-normal distribution. T test or the Wilcoxon rank-sum test was used to compare differences among 2 groups for normally and non-normally distributed variables, respectively. Statistical significance was set at two tailed p < 0.05. Statistical analyses were performed using IBM SPSS Statistics for Windows, version 22.0 (IBM Corp., Armonk, N.Y., USA) and R software, version 3.5.3 (The R Foundation, Vienna, Austria).

## Conclusions

In summary, we demonstrate novel differences in LA function between CA subtypes despite similar LA geometry. Our findings of more impaired LA function in ATTR may offer insight into higher AF burden in these patients.
